# Point of Care Ultrasound First: An Opportunity to Improve Efficiency for Uncomplicated Pregnancy in the Emergency Department

**DOI:** 10.24908/pocus.v6i1.14762

**Published:** 2021-04-22

**Authors:** Sara Urquhart, Kendall Stevens, Mariah Barnes, Matthew Flannigan

**Affiliations:** 1 Spectrum Health -Michigan State University College of Human Medicine Grand Rapids, MI

**Keywords:** POCUS, bedside ultrasonography, pregnant, IUP, throughput, length-of-stay

## Abstract

**Introduction: **Research suggests emergency providers using point-of-care ultrasound (POCUS) to confirm an uncomplicated intrauterine pregnancy (IUP) can decrease emergency department (ED) length of stay (LOS) compared to a radiology department ultrasound (RADUS). The objective of this study was to compare the time to diagnosis and LOS between POCUS and RADUS patients. **Methods: **This was a retrospective study at one urban medical center. A standardized tool was used to abstract data from a random sample of pregnant patients diagnosed with uncomplicated IUP between January 2016 and December 2017 at a single tertiary care medical center. Microsoft Excel 2010 software was used to measure time intervals, prepare descriptive statistics, and perform Mann-Whitney U tests to compare differences. **Results: **A random sample of 836 (36%) of the 2,346 emergency department patients diagnosed with an IUP between 8-20 weeks’ gestation during the study period was evaluated for inclusion. Three hundred sixty-six met inclusion criteria and were included in the final analysis. Patients were divided into 2 groups based on which type of ultrasound scan they received first: POCUS (n=165) and RADUS (n=201). Patients who received POCUS were found to have an IUP identified in an average of 48 minutes (95% CI, 43 to 53), while the RADUS group’s mean time to diagnosis was 120 minutes (95% CI 113 to 127) with a difference of 72 minutes (95% CI, 63 to 80; p<0.001). The mean LOS for patients who received POCUS was 132 minutes (95% CI, 122 to 142), while that of the RADUS group was 177 minutes (95% CI 170 to 184) with a difference of 45 minutes (95% CI 32 to 56; p<0.001). The study is limited by its single-center, retrospective design and by lack of blinding of data abstractors. **Conclusion: **Pregnant emergency department patients diagnosed with an uncomplicated IUP between 8-weeks and 20-weeks’ gestation had statistically significant reduction in time to diagnosis and disposition from the ED if assessed with POCUS as compared to RADUS.

## Background

According to the American College of Emergency Physicians (ACEP) clinical guidelines, emergency physicians should obtain a pelvic ultrasound for symptomatic pregnant patients with any ß-hCG level to confirm an intrauterine pregnancy (IUP) 1. However, sending a patient for a radiology department performed ultrasound and awaiting a radiologist interpretation can be time consuming, especially in hospitals that do not employ an ultrasound technician 24 hours per day. In the past two decades, mounting research suggests emergency providers can accurately use point-of-care ultrasound (POCUS) to confirm an uncomplicated intrauterine pregnancy (RADUS) while decreasing emergency department (ED) length of stay (LOS) 2, 3, 4, 5, 6, 7, 8, 9, 10. The goal of our study was to measure and compare the time interval from ED arrival to ultrasound diagnosis and the LOS between patients who received initial POCUS or RADUS in our emergency department.

## Methods

The study was approved by the hospital Institutional Review Board (Study ID: 2019-059). This was a retrospective study comparing time from ED arrival to ultrasound interpretation and time from ED arrival to ED departure between patients who received POCUS and patients who had a radiology department ultrasound (RADUS). The study took place at an urban tertiary care academic medical center with 99,900 ED visits per year during the study period. POCUS exams were performed by emergency medicine (EM) residents with attending supervision, ultrasound fellows, and board-certified or board-eligible EM attending physicians. All examinations were completed using transabdominal views in a structured format using Zonare Ultra or Pro models with either a P4-1c or C6-2 transducer (Mindray, Mountain View, CA) and digitally archived in QPath (Telexy Healthcare, Maple Ridge, British Columbia, Canada).

The study examined ED patients with an ultrasound confirmed IUP between January 2016 and December 2017. An honest broker service was used to generate a list of ED patients with international classification of disease (ICD) codes for intrauterine pregnancy of 8-20 weeks. The list of patients was randomized and a sample of 36% of the patients was selected for inclusion analysis. Only pregnant ED patients ≥18 years who received their initial diagnosis of an uncomplicated IUP between 8-20 weeks gestation were included in the final analysis. Uncomplicated was defined by the lack of exclusion criteria. Patients were excluded if they were taking fertility treatments, had BMI ≥40, had a known uterine anomaly, received an extended workup for a non-obstetric complaint, were admitted, were transferred in from an outside facility, had a new secondary diagnosis, or had prior ultrasound confirmed IUP. Patients with missing timestamp data were excluded from the final analysis as well. Missing data was not imputed. An ultrasound diagnosis of an IUP was defined by visualization of an intrauterine yolk sac or fetal pole. Arrival time was defined by the timestamp from the first recorded vital sign. Ultrasound time was defined either by the POCUS acquisition time or the radiologist interpretation timestamp, which are both digitally recorded. Departure time was defined by the patient departure timestamp. There were consistent workflows in place during the study period by which these times were recorded in the electronic medical record (EMR). A standardized abstraction tool (Appendix 1) was used to guide data collection in a coded, systematic fashion. Each patient’s EMR was individually analyzed to abstract data points and timestamps for ED arrival, ultrasound scan(s), and ED departure. Data abstractors were initially trained and periodically audited for adherence to data collection protocols and were not blinded to the study hypothesis. In the case of patients with questionable inclusion or exclusion criteria, the record was reviewed independently by another researcher. In the case of disagreement, the record was reviewed for inclusion via research team conference, however interobserver reliability ratings were not performed. The time intervals were measured and compared between patients receiving POCUS and RADUS and descriptive statistics were prepared. Mann-Whitney U tests were performed to examine differences between the two groups with a p-value of <0.05 considered statistically significant. All calculations were performed using Microsoft Excel 2010 software.

## Results

A random sample of 836 (36%) of the 2,346 emergency department patients diagnosed with IUP between 8-20 weeks’ gestation during the study period were evaluated for inclusion. Three hundred sixty-six met inclusion criteria and were included in the final analysis. Patients were divided into 2 groups for analysis based on which type of ultrasound scan they received first: POCUS (n=165) and RADUS (n=201). There were 8 patients who received POCUS first, followed by a RADUS that were included in the POCUS group for analysis. The average ages of the POCUS and RADUS groups were 25 and 26, respectively. Out of 366 included patients, 163 (45%) had vaginal bleeding and 264 (72%) reported abdominal pain. Other common complaints were vaginal discharge, nausea, and dysuria. When comparing the presenting complaints of abdominal pain between the POCUS and RADUS groups, 115 (70%) POCUS patients and 151 (75%) RADUS patients had a documented complaint of abdominal pain. A complaint of vaginal bleeding was recorded in 68 (41%) of POCUS patients and 92 (46%) of RADUS patients. 

Patients who received POCUS were identified to have an IUP in an average of 48 minutes (95% CI, 43 to 53), while the RADUS group’s mean time to diagnosis was 120 minutes (95% CI 113 to 127) with a difference of 72 minutes (95% CI, 63 to 80; p<0.001) (Table 1). 

The mean LOS of patients who received POCUS was 132 minutes (95% CI, 122 to 142), while that of the RADUS group was 177 minutes (95% CI 170 to 184) with a difference of 45 minutes (95% CI 32 to 56; p<0.001) (Table 1).

**Table 1:** Comparison of time intervals for POCUS and RADUS groups

		**POCUS (n=165)**	**RADUS (n=201)**
**Time interval between ED arrival and first scan (minutes)**	Mean	48 (CI 43-53)	120 (CI 113-127)
	Range	0-150	20-225
	Difference	72 (CI 63-80)	
	p-value	<0.001	
**Time interval between ED arrival and departure (minutes)**	Mean	132 (CI 122-142)	177 (CI 170-184)
	Range	32-283	63-354
	Difference	45 (CI 32-56)	
	p-value	<0.001	

## Discussion

This study is limited by its single-center, retrospective design which decreases generalizability and prevents group randomization to control for confounders. A systematic review performed by Beals et al in 2019 of 6 retrospective studies similar to this one demonstrated a mean decrease of 73.8 minutes in LOS in POCUS patients compared to RADUS patients 4. A randomized controlled trial undertaken by Morgan et al found an average reduction of 49 minutes in time to diagnosis and 20 minutes in LOS for POCUS patients and provides an excellent design framework for future studies of this kind 5. Our study, with an average decreased time to diagnosis of 72 minutes and average reduced LOS of 45 minutes, provides validation of prior findings. Variables such as institution-specific workflows, availability of ultrasound machines, POCUS skill level, and type of provider (fellowship-trained physician, non-fellowship trained physician, or advanced practice provider) may influence time to diagnosis and LOS metrics. 

In our study and all referenced studies, there were a subgroup of patients who received POCUS, but needed additional radiology department ultrasound services. Our collaboration with the radiology department is critical to improving care and efficiency for our pregnant patients. Although POCUS has a nearly a 100% specificity rate for IUP, the radiology department scan is more comprehensive and a radiologist’s interpretation can be helpful in the evaluation of additional pathology. For all 8 patients in our study who received POCUS followed by RADUS, we noted that the physician wanted additional data that was not well-characterized with POCUS.

Other previous research has demonstrated additional benefits of using POCUS for patients with uncomplicated IUP, including increased accuracy, decreased length of stay (LOS), and improved patient satisfaction scores 2, 3, 4, 5, 6, 7, 8, 9. For example, Blaivas et al noted the reduced cost related to shorter length of stay and elimination of the need for transport out of the emergency department 10. Despite growing awareness that POCUS has many patient-centered benefits, barriers such as lack of training and lack of access to equipment continue to prevent this from becoming a widely accepted practice. While it has been demonstrated that POCUS decreases time to diagnosis and LOS of our pregnant ED patients, additional work is needed to ensure pregnant ED patients everywhere are able to access this approach.

## Conclusions

Pregnant emergency department patients diagnosed with an uncomplicated IUP between 8 and 20 weeks gestation had statistically significant lower times to diagnosis and departure if assessed with POCUS as compared to RADUS. Based on the agreement of our results with past research, we conclude that, for the confirmation of uncomplicated IUP in the ED setting, POCUS reduces time to diagnosis and LOS compared to RADUS.

## Disclosures

The authors do not have any conflicts of interest to report.

## Supplementary Material 

Appendix I
