# Supplementary material for: Point of Care Ultrasound First: An Opportunity to Improve Efficiency for Uncomplicated Pregnancy in the Emergency Department
Source: POCUS J. 2021 Apr 22;6(1):42–4. doi: 10.24908/pocus.v6i1.14762 (PMC9979936; doi:10.24908/pocus.v6i1.14762)
Supplement: Appendix I [file pocusj-06-14762-s001.pdf]

POCUS DIAGNOSIS OF IUP DATA COLLECTION														
**Study ID**	**Age**	**ED Presentation Time**	**Prior US confirmation of IUP for same pregnancy?**	**Vaginal Bleeding**	**Abdominal pain**	**Other**	**POCUS**	**Time of POCUS**	**Result of POCUS**	**Radiology US**	**Time of Rad US**	**Result of US**	**Arrival to First Study**	**Arrival to Dispo**
